# Research and Application of Ga-Based Liquid Metals in Catalysis

**DOI:** 10.3390/nano15151176

**Published:** 2025-07-30

**Authors:** Yu Zhang, Ying Xin, Qingshan Zhao

**Affiliations:** State Key Laboratory of Heavy Oil Processing, College of Chemistry and Chemical Engineering, China University of Petroleum (East China), Qingdao 266580, China; s24030086@s.upc.edu.cn (Y.Z.); z24030047@s.upc.edu.cn (Y.X.)

**Keywords:** liquid metals, Ga-based catalysts, electrocatalysis, thermal catalysis, photocatalysis

## Abstract

In recent years, Ga-based liquid metals have emerged as a prominent research focus in catalysis, owing to their unique properties, including fluidity, low melting point, high thermal and electrical conductivity, and tunable surface characteristics. This review summarizes the synthesis strategies for Ga-based liquid metal catalysts, with a focus on recent advances in their applications across electrocatalysis, thermal catalysis, photocatalysis, and related fields. In electrocatalysis, these catalysts exhibit potential for reactions such as electrocatalytic CO_2_ reduction, electrocatalytic ammonia synthesis, electrocatalytic hydrogen production, and the electrocatalytic oxidation of alcohols. As to thermal catalysis, these catalysts are employed in processes such as alkane dehydrogenation, selective hydrogenation, thermocatalytic CO_2_ reduction, thermocatalytic ammonia synthesis, and thermocatalytic plastic degradation. In photocatalysis, they can be used in other photocatalytic reactions such as organic matter degradation and overall water splitting. Furthermore, Ga-based liquid metal catalysts also exhibit distinct advantages in catalytic reactions within battery systems and mechano-driven catalysis, offering innovative concepts and technical pathways for developing novel catalytic systems. Finally, this review discusses the current challenges and future prospects in Ga-based liquid metal catalysis.

## 1. Introduction

Traditional solid-phase catalysts have enabled significant progress in many catalytic reactions due to their diverse and tunable components, forms, and structures. However, these solid-phase catalysts are subject to limitations, such as deactivation via coking [1]. These challenges have stimulated extensive research and exploration into innovative catalysts. Among numerous potential candidate materials, liquid metals have garnered significant attention as an emerging research focus owing to their unique properties [2]. Generally, liquid metals refer to metals or alloys that remain liquid at relatively low temperatures (typically from room temperature to 300 °C). Composed predominantly of post-transition metal elements (such as Ga, In, Sn, and Bi), these systems can dissolve small amounts of transition or precious metal solutes, forming low-melting-point alloys with unique catalytic properties. The solubility of added elements in liquid metals is strongly dependent on the temperature, and follows the general relationship described in Equation (1) [3]. Research demonstrates that combining liquid metals with specific solute metals significantly modifies their physicochemical characteristics, thereby enhancing their catalytic performance. When the composition of a solute metal exceeds its solubility limit in a liquid metal, the liquid metal transforms from a fully disordered liquid state to a solid-in-liquid metal colloid. The regulation of this colloidal solid provides additional degrees of freedom for manipulating the properties and performance of liquid metal alloys. The solid colloidal particles may offer additional catalytic interfaces or serve as reservoirs for catalytically active solutes, which are released upon heating. Consequently, establishing structure–property correlations for liquid metal colloids incorporating solid metallic entities and intermetallic compounds is crucial for developing novel catalytic materials [4,5]. Furthermore, metal clusters have been observed in liquid metal systems. Eutectic liquid metals are not the traditionally perceived “homogeneous monoatomic fluids” but, rather, soft matter possessing dynamically nanostructured features. Eutectic alloys such as EGaIn and Galinstan form stable nanoscale clusters in the liquid state, a structure that remains stable even at elevated temperatures. These clusters are not rigid particles but dynamic soft domains composed of different elements. The cluster structures may act as active sites influencing dissolution, nucleation, and catalytic pathways; variations in atomic spacing and electronic structure within domains of different elements can modulate reaction energy barriers. The formation of metallic clusters in liquid metals strictly depends on the eutectic composition, and their stability is maintained at high temperatures. This discovery establishes a structural foundation for designing high-performance liquid metal catalysts (e.g., by tailoring cluster size/composition to optimize active sites) [6,7].

(1) $$\log_{10}C=A-\frac{B}{T}$$

(here, C is the solubility in atomic percent (at%), A and B are experimentally determined constants, and T is the absolute temperature in K).

In the 19th and 20th centuries, Hg became a staple in laboratories and various industrial processes. However, the toxicity of Hg limits its development [8]. Among the liquid metals, low-toxicity Ga has gained prominence as a research focus in catalysis and liquid support materials due to its distinctive characteristics such as a low melting point, excellent metal solubility, and a dynamically self-healing oxide layer [9]. The melting point is a fundamental factor that determines the temperature range at which it exists in liquid form and affects its stability and application [10]. The melting point of pure Ga is 29.8 °C, and it can be further reduced through alloying. For instance, the melting point of GaIn alloy is approximately 15.7 °C. These alloys remain liquid over a wide temperature range (typically −20 °C to several hundred °C), providing a dynamic liquid interface for catalytic reactions (the melting points of common liquid metals and alloys are summarized in Table 1 [11,12,13,14,15]). Metal solubility represents another critical property of Ga-based liquid metals. This property enables the dissolution of diverse metallic elements to form alloys, providing the possibility of precisely regulating their catalytic performance (such as activity and selectivity) through metal doping strategies [3]. Although emerging solid catalysts such as nano-oxides and doped carbon can similarly modulate their properties through doping or supporting other metals, the solubility of Ga-based liquid metals enables a more homogeneous bulk alloy environment and grants dynamic compositional evolution capability at the interface. Dissolved metal atoms can achieve atomic or near-atomic dispersion within the liquid matrix, forming a uniform distribution of active sites analogous to the concept of single-atom catalysts (SACs), with the distinction that the support is a liquid metal possessing a fluid nature.

In terms of thermophysical properties, Ga-based liquid metals exhibit superior thermal conductivity, with a thermal conductivity (~33 W/(m·K)) significantly exceeding that of water (by over 40 times). Combined with their higher density, their volumetric heat capacity also far surpasses that of water [16]. Their thermal conductivity is also substantially higher than that of nanostructured oxides [17]. In contrast, although doped carbon materials possess high theoretical thermal conductivity (e.g., the thermal conductivity of a single graphene layer is 5300 W/(m·K)), the thermal conductivity of their composites is typically much lower than the theoretical value for monolayer graphene, susceptible to interfacial thermal resistance, and lacks dynamic tunability [18]. These characteristics jointly endow Ga-based liquid metals with efficient heat transfer and dissipation capabilities, which help maintain the uniformity of system temperature in catalytic reactions, reducing local hotspots. In terms of chemical stability, nanostructured oxides are relatively chemically stable and generally do not readily undergo chemical reactions with other substances under ordinary conditions. While doped carbon materials also exhibit relatively high chemical stability, their catalytic activity (particularly electrocatalytic activity) and electrical conductivity are heavily dependent on the type and concentration of dopant elements. Furthermore, their surfaces are prone to deactivation due to the adsorption of contaminants [19,20,21]. When exposed to air or oxygen, Ga-based liquid metals spontaneously form an ultrathin (~1–3 nm) amorphous Ga_2_O_3_ surface layer [22]. Although this renders its bulk less chemically stable than nanostructured oxides or doped carbon, this oxide layer precisely constitutes its functional core. This oxide layer passivates the bulk metal against further oxidations and modifies surface tension and interaction between the Ga-based liquid metal and the substrate, facilitating the liquid metal to adhere to the surrounding environment. In addition, the oxide layer undergoes localized fracture and regeneration under shear forces, electric fields, or chemical reduction, continuously exposing fresh active metal surfaces. This dynamic self-repairing mechanism effectively enhances catalyst longevity and mitigates carbon deposition. Meanwhile, the surface oxide layer and the liquid metal substrate beneath it can manipulate the oxide species on the surface through controlled electron transfer [23,24].

This review systematically summarizes the categories and synthesis strategies of Ga-based liquid metal catalysts and their application advances in diverse catalytic contexts, including electrocatalysis (such as electrocatalytic CO_2_ reduction, electrocatalytic ammonia synthesis, electrocatalytic hydrogen production, and electrocatalytic oxidation of alcohols), thermocatalysis (such as alkane dehydrogenation, selective hydrogenation, thermocatalytic CO_2_ reduction, thermocatalytic ammonia synthesis, and plastic degradation), photocatalysis (organic matter degradation and overall water splitting), electrode interface catalysis in batteries, and mechanically driven catalytic reactions. Finally, this review elaborates on the current challenges and outlines the prospects in liquid metal catalysis.

## 2. Categories and Fabrication of Ga-Based Liquid Metal Catalysts

Owing to its unique ability to dissolve metals, liquid Ga can form composites with various metals, resulting in bimetallic catalysts, polymetallic catalysts, or supported catalytically active liquid metal solution (SCALMS) catalysts. Due to synergistic effects, bimetallic and polymetallic catalysts exhibit superior catalytic activity as compared to their monometallic counterparts [25].

### 2.1. Bimetallic Catalysts

Liquid Ga, leveraging its unique metal solubility and dynamic fluidity, enables the fabrication of diverse bimetallic catalytic systems through precisely controlled synthesis strategies, including mechanical alloying, electrochemical internalization, and ultrasonic dispersion. These approaches facilitate combination with matrix metals (In, Sn), transition metals (Cu), and noble metals (Pt, Pd), where the selection of synthesis routes is determined by the inherent properties of each metal category.

Ga can form low melting point alloys with metals such as In [26], Sn [27], etc. The melting point of the alloy varies depending on its composition and proportion. Adjusting the Ga/In ratio enables the synthesis of GaIn alloys with tunable compositions and melting points. EGaIn specifically refers to the GaIn alloy with a composition of 75 wt% Ga and 25 wt% In, which is a low melting point eutectic alloy [28]. 75 wt% Ga and 25 wt% In were placed in a high temperature resistant container, heated and melted at 200 °C, stirred until evenly mixed, and then naturally cooled to form the eutectic alloy [29]. Similarly, GaSn can be synthesized in this way.

Ga enables the fabrication of bimetallic catalysts with transition metals. The Ga–Cu bimetallic catalyst can be fabricated via the following two approaches: the mechanical milling alloying method and the deposition-internalization method. Cu and Ga were mechanically ground at 400 °C for 1 h to ensure that Cu was completely dissolved in Ga. The process was conducted under a nitrogen atmosphere to prevent oxidation. The resulting alloy was quenched in a −89 °C freezer, cured, and stored solid (melting point ≈ 25 °C). Next, the prepared alloy was added to a container containing a high-temperature solvent (1-ethyl-3-methylimidazole-tetrafluoborate) and ultrasonically treated at 300 °C with a power of 300 W for 30 min using a probe-type ultrasonic instrument. After that, the ionic liquid solvent was removed through steps such as cooling, dilution, and vacuum filtration, and the alloy was re-dispersed in a 90% ethanol aqueous solution. The Cu-Ga liquid metal catalyst can be obtained after ethanol removal [30]. A flowable and renewable CuGa–LM electrode was prepared by the deposition internalization method. Firstly, in the CuSO_4_-H_2_SO_4_ electrolyte, Cu^2+^ was electrodeposited onto Ga at −2.5 V for 20 s using a copper wire inserted into Ga as the working electrode. Subsequently, a −4 V voltage was applied for 3 s to trigger the internalization effect of the liquid metal, absorbing the copper deposited on the surface into the Ga body, and the reaction formed CuGa_2_. By repeating the cycle to internalize different amounts of copper into Ga and then annealing at 180 °C for 2 h to remove residual stress and defects, the CuGa–LM catalyst loaded on a flowing Ga substrate was finally obtained [31].

**Figure 1:**
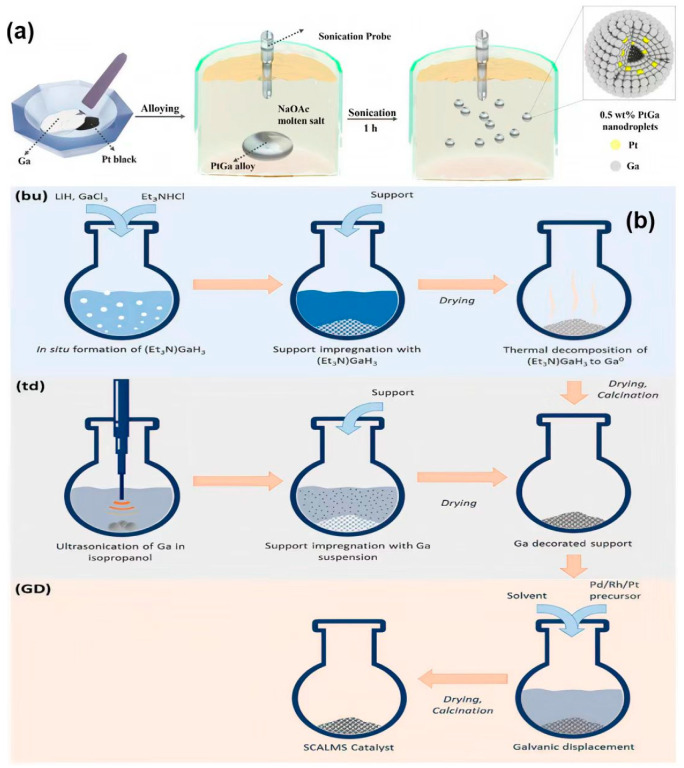
(**a**) Schematic illustration of Pt–Ga alloy and nanodroplets synthesis [34]. (**b**) Bottom-up (bu) synthesis of Ga-functionalized supports via thermal decomposition of triethylamino-gallane; top-down (td) functionalization of supports with Ga using ultrasonic treatment of Ga nuggets in isopropanol; (GD) deposition of the active metal by galvanic displacement, yielding the final solution [40].

The liquid Ga can be used to fabricate high-performance bimetallic catalysts through a combination with noble metals (such as Pt and Pd). The liquid Ga can achieve the atomic-level dispersion of Pt and modulate the electron structure through charge transfer, solving the problems of agglomeration, poisoning, and the low atomic efficiency of traditional Pt catalysts. Liquid Ga-based catalysts were prepared by ultrasonic dispersion technology. Ga was dispersed in isopropyl alcohol at approximately 50 °C to form an emulsion. Different amounts of H_2_Cl_6_Pt·6H_2_O solution were introduced into the above emulsion by the impregnation method. After stirring at room temperature for 2 h, the mixture was dried in a vacuum oven to obtain catalysts with different Pt loading amounts. Compared with traditional catalysts that use Ga_2_O_3_ or Ga_2_(NO_3_)_3_·6H_2_O as precursors, this method does not require calcination or reduction steps, avoids the problem of Pt particle agglomeration, and achieves the atomic-level dispersion of Pt in Ga [32]. The liquid Ga–Pt alloy catalyst was prepared by the pre-saturation method. Pt and Ga were mixed and continuously heated at 400 °C for 4 h to ensure the complete dissolution of Pt. The dissolved Ga–Pt alloy was cooled to the required experimental temperature. When in use, it is removed from the top of the mixture to prevent the re-solidified Pt portion that has exceeded the solubility limit from being taken out [33]. Alternatively, Pt–Ga nanodroplets could be prepared by the high temperature ultrasonic method. Pt black powder and Ga metal were mechanically mixed in a mortar and pestle for approximately 60 min to form a uniform alloy. The alloy was placed in molten NaOAC and subjected to high-intensity ultrasonic treatment at 400 °C for 1 h using a probe ultrasonic instrument to synthesize nanodroplets (Figure 1a) [34]. Moreover, the Ga–Pd intermetallic compound prepared by a two-step synthesis method of co-reduction and annealing, using GaCl_3_ and Pd(acac)_2_ as metal sources, was co-reduced with the metal ion precursors using LiHBEt_3_ in tetrahydrofuran. Pd nanoparticles were formed first without the need for additional stabilizers, and then Ga^3+^ was catalytically reduced on the Pd surface. After annealing the incompletely alloyed Pd–Ga intermediate products, uniform GaPd and GaPd_2_ intermetallic compounds were formed, and the Ga–Pd nanocatalyst was obtained. To further enhance the activity of the catalyst, nanoparticles can be deposited in α-Al_2_O_3_ [35].

### 2.2. Polymetallic Catalysts

Polymetallic catalysts refer to composite catalytic materials composed of three or more metallic elements, where synergistic catalytic activity arises from electronic and geometric effects between the constituents. Among these, Ga-based eutectic alloys serve as important carriers for polymetallic catalysts due to their liquid matrices, enabling dynamic regulation of active sites.

The Ga_75_In_25_Sn_10_ (Galinstan) eutectic alloy, composed of 75 wt% Ga, 25 wt% In, and 10 wt% Sn, exhibits a melting point of −19 °C and remains liquid at room temperature [12]. The constituent metals were placed in a high temperature resistant container, heated to 200 °C to melt, stirred until homogeneous, and subsequently cooled naturally to form the Galinstan eutectic alloy [36].

EGaIn and Galinstan are the most commonly used Ga-based liquid alloys. Studies demonstrate that doping with other metal elements can significantly enhance their catalytic performance. For instance, doping Cu into EGaIn enables efficient hydrogen production. By mixing Cu and EGaIn in a specific proportion and grinding them by solvent-free ball milling, the uniform dispersion and dissolution of Cu in EGaIn could be achieved. X-ray diffraction (XRD) analysis indicated that the Ga_2_Cu intermetallic compound was formed in the Cu–EGaIn system. This catalyst demonstrated excellent performance in the in situ hydrogen production reaction with high efficiency and mild conditions [37]. Similarly, when Cu was dissolved in liquid Galinstan alloy, the resulting catalyst exhibited dynamic adaptive structural characteristics and was able to spontaneously adjust its configuration under reaction conditions to optimize the catalytic process [38]. Ce-doped Galinstan can be used as an anti-carbon deposit CO_2_ reduction catalyst. Ce was doped into Galinstan (usually by mechanical grinding to give the mixture a smooth and reflective appearance) to form an LMCe catalyst. In this system, Ce exists in the form of nanoparticles and forms a thin CeO_2_ layer on the surface of the liquid metal. These cerium oxide layers and Ce nanoparticles together constitute the catalytic active sites. When this catalyst is used for the electrocatalytic reduction of carbon dioxide, the carbonaceous products generated will not adhere to the electrode surface through van der Waals forces, thereby effectively avoiding the carbon deposit and deactivation of the catalyst. This anti-carbon deposit property enables the LMCe catalyst to maintain stable catalytic activity during long-term operation [39].

### 2.3. Supported Catalytic Active Liquid Metal Solution (SCALMS) Catalysts

In recent years, SCALMS catalysts have been proven to be a highly successful concept, integrating the advantages of heterogeneous catalysis and molecular catalysis. SCALMS catalysts are composed of active metals (such as Pd, Rh, Pt, Ni) and low-melting-point metals (such as Ga, In, Sn) in the liquid metal solution and are deposited on porous carrier materials. This type of catalyst exhibits high activity and anti-carbon deposit ability in the dehydrogenation reaction of alkanes. The preparation methods of supported catalysts can be divided into the top-down approach and the bottom-up approach, as shown in Figure 1b. The method of breaking bulk metal Ga into tiny droplets through physical methods (such as ultrasound) and depositing them on the carrier is called the top-down approach. The bottom-up approach is to generate and decompose metal Ga nanoparticles in situ through Et_3_NGaH_3_ and load them on the carrier [40].

Using Et_3_NGaH_3_ and [NH_4_]_2_[PdCl_4_] as metal precursors, macroporous glass was immersed in an ether solution containing Et_3_NGaH_3_. Following solvent removal under vacuum, the resulting Ga-loaded support material was brought into contact with an aqueous solution of [NH_4_]_2_[PdCl_4_]. The GaPd–SCALMS catalysts were prepared by introducing Pd into the Ga-loaded support through the electro-displacement reaction [41]. The high solubility of Pd atoms within the liquid Ga matrix facilitates their uniform distribution and prevents aggregation into nanoparticles. Furthermore, the continuous flow at the liquid surface ensures the dynamic exposure of catalytically active sites. The GaRh–SCALMS catalyst can also be synthesized by this method. Et_3_NGaH_3_ and RhCl_3_·3H_2_O were used as metal precursors. Al_2_O_3_ was immersed in the ethereal Et_3_NGaH_3_ solution to yield a Ga-loaded Al_2_O_3_ support. Subsequently, an aqueous RhCl_3_·3H_2_O solution was added, and Rh was introduced onto the Ga-loaded support via electro-displacement [42]. Similarly, GaPt–SCALMS can be prepared by the same method using Et_3_NGaH_3_ and Pt(NO_3_)_2_ as metal precursors [43]. Alternatively, different Ga dispersion strategies can be adopted. Ga was dispersed in isopropyl alcohol solution using an ultrasonic instrument to form a fine emulsion, and the required support (Al_2_O_3_, SiO_2_, and SiC) was added to the fine emulsion to achieve loading. Adding H_2_Cl_6_Pt·6H_2_O to the isopropyl alcohol solution containing the Ga-modified support, evaporating the solvent to obtain the product, and calcining it overnight at 450 °C can also yield GaPt–SCALMS [44].

## 3. Application of Ga-Based Liquid Metals in Catalysis Reactions

### 3.1. Application of Ga-Based Liquid Metals in Electrocatalysis

Ga-based catalysts leverage their superior electrical conductivity and thermal conductivity to enable rapid electron and heat transport during electrocatalytic processes. This characteristic significantly enhances electrochemical reaction kinetics while maintaining system stability through efficient heat dissipation that prevents localized overheating, thus systematically improving catalytic efficiency. Currently, such catalysts demonstrate application potential in diverse electrocatalytic reactions such as CO_2_ reduction, ammonia synthesis reaction, hydrogen evolution reaction, and the electrocatalytic oxidation of alcohols.

#### 3.1.1. Electrocatalytic CO_2_ Reduction

Since the Industrial Revolution, humanity’s heavy reliance on fossil fuels has led to a year-on-year increase in the concentration of CO_2_ in the global atmosphere, which has caused problems such as the intensification of the greenhouse effect, global temperature rise, and ocean acidification [45]. The conversion of CO_2_ into carbon or value-added chemicals represents an effective approach to reducing CO_2_ concentration and addressing environmental and energy issues. Ga-based liquid metal catalysts have demonstrated enormous potential in the field of electrocatalytic CO_2_ reduction owing to their unique properties. By optimizing the composition, structure, and preparation process of the catalysts, their catalytic performance can be significantly enhanced, offering possibilities for the efficient conversion and utilization of CO_2_.

**Figure 2:**
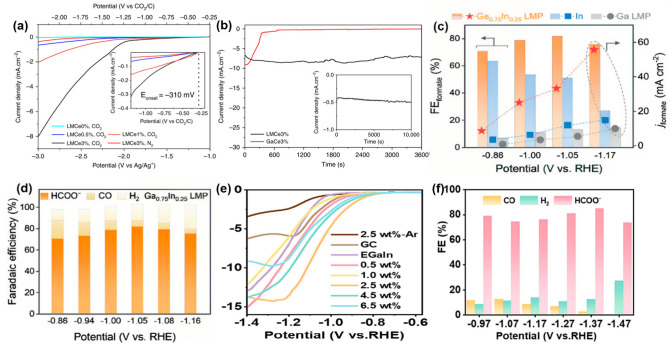
(**a**) Linear sweep voltammetry (LSV) was performed on Galinstan electrodes to investigate the electrochemical response at varying concentrations of Ce [39]. (**b**) Chronoamperometric data for solid Ga and liquid Ga doped with 3 wt% Ce were recorded at a constant potential of −3 V vs. Ag/Ag^+^ within a CO_2_-saturated electrolyte [39]. (**c**) Polarization curves of Ga_0.75_In_0.25_ LMP, In micropowders, and Ga LMP in CO_2_-saturated 0.5 m KHCO_3_ in an H-cell [50]. (**d**) Potential-dependent formate Faradaic efficiency and partial current density of Ga_0.75_In_0.25_ LMP, In micropowders, and Ga LMP [50]. (**e**) LSV curves of U-hybridized EGaIn capsules in 0.5 M KHCO_3_ electrolyte for CO_2_ reduction. *J* (mA cm^−2^) is the absolute current normalized by electrode area [51]. (**f**) Faradaic efficiencies for target products of CO, H_2_, and HCOO^−^ [51].

The direct conversion of CO_2_ into carbon represents a highly effective pathway for reducing CO_2_ concentrations. Esrafilzadeh et al. [39] reported a Ce-doped Galinstan alloy that reduced CO_2_ to layered amorphous carbon nanosheets. The experiment used a dimethylformamide solution containing 100 mM tetrabutylammonium hexafluorophosphate and 2 M H_2_O as the electrolyte (freshly prepared before each use). The current density was observed to correlate with Ce content. The best-performing LMCe 3% electrode achieved CO_2_ reduction to carbon nanosheets (thickness ~3 nm) at a low onset potential of −310 mV vs. CO_2_/C, with a Faradaic efficiency of 75% (Figure 2a). While the room-temperature solid GaCe3% electrode demonstrated comparable initial CO_2_ electrolysis activity to its liquid counterpart, rapid carbon deposition-induced deactivation occurred. This performance decay highlights the liquid electrode’s necessity for sustained operation. (Figure 2b). The current density remained stable after continuous electrolysis for 20 h (−3 V vs. Ag/Ag^+^). In situ Raman spectroscopy and X-ray photoelectron spectroscopy (XPS) analysis revealed that Ce drove the catalytic process through a Ce^2+^/Ce^0^ redox cycle, with Ce nanoparticles embedded within the liquid metal enhancing catalyst activity. Lörch et al. [46] addressed the challenge of incorporating cerium oxide layers by optimizing the mechanical alloying preparation process of the catalyst, significantly improving its reproducibility and performance. Babikir et al. [47] utilized dispersed liquid metal Ga combined with dielectric barrier discharge (DBD) plasma technology to rapidly convert CO_2_ into a solid carbon/GaOOH nanocomposite. Raman and XRD experiments confirmed the formation of amorphous activated carbon from CO_2_ after plasma treatment.

The conversion of CO_2_ into value-added chemicals (such as CO and formic acid) represents an ideal strategy to address the environmental issues and energy challenges arising from continuously increasing global CO_2_ levels [48]. Liquid metal catalysts demonstrate potential in the electrocatalytic reduction of CO_2_ to formic acid due to their unique properties. The study by Liu et al. [49] revealed the significant influence of the solid–liquid phase transition in Ga–Sn (Ga_0.916_Sn_0.084_) and Ga–In (Ga_0.835_In_0.165_) alloys on their performance for the CO_2_ reduction reaction to formic acid. The reaction was performed in a dual-compartment H-type electrolytic cell using an electrolyte containing 0.1 M NaCl and 25 mM NaOH (pH ≈ 7), under continuous CO_2_ bubbling (150 mL/min) and within a potential range of −0.76 to −1.26 V (vs. RHE) for electrocatalytic CO_2_ reduction. The research indicated that the phase transition process could trigger dynamic restructuring of the catalyst’s atomic and electronic structures, thereby significantly affecting its catalytic performance. Following prolonged operation at 8 °C in the solid state, the electrode surface formed a dark passivation layer due to oxidation and electrolyte adsorption, leading to aggravated hydrogen evolution side reactions and a sharp decline in formate selectivity. Subsequently, merely raising the system temperature to 23 °C caused the alloy to melt into the liquid phase. Its atomic-level fluidity promoted the instantaneous exfoliation of the surface oxide layer, allowing the Sn/In active centers to uniformly redisperse in a single-atom form. This restored and optimized the electron density and the binding strength with the *OCHO intermediate. Electrocatalysis at −1.26 V vs. RHE for 25 min restored the metallic luster and fully recovered >95% of the formate Faradaic efficiency, achieving “self-healing” regeneration. This process could be cycled repeatedly without activity loss. Furthermore, Huang et al. [50] fabricated compositionally tunable binary Ga_1-x_In_x_ and Ga_1−x_Sn_x_ liquid–metal alloys via an alloying strategy and systematically investigated their performance in the electrocatalytic reduction of CO_2_ to formate. The reaction was conducted in a dual-compartment H-cell, with the cathode compartment containing a 0.5 M KHCO_3_ solution saturated with CO_2_ and applied potentials ranging from −0.8 to −1.2 V (vs. RHE). The performance of Ga_0.75_In_0.35_ LMP was studied as an exemplary catalyst for formate production in the electrocatalytic CO_2_ reduction reaction. By contrast, Ga_0.75_In_0.35_ LMP exhibited significantly higher formate selectivity, with an average Faradaic efficiency exceeding 70% across the potential window. The peak formate Faradaic efficiency reached 82% at −1.05 V, representing a considerable improvement compared to Ga LMP and In. Comparing the formate partial current densities of the three samples at different working potentials (Figure 2c,d), the formate partial current density for Ga_0.75_In_0.25_ was 55 mA cm^−2^ at −1.17 V, approximately 4–6 times higher than that of Ga LMP and In powder. Ga_1−x_Sn_x_ also demonstrated excellent catalytic performance for formate production in the electrocatalytic CO_2_ reduction reaction.

EGaIn liquid metal nanodroplets can be used as bifunctional materials, which can efficiently adsorb uranyl ions (UO_2_^2+^) in aqueous solutions and catalyze the electrochemical reduction of CO_2_ to formic acid. Zhai et al. [51] prepared EGaIn droplets (with an average particle size of 134 nm) by an ultrasonic method, which showed a uranium adsorption capacity as high as 237 mg g^−1^, and simultaneously reduced U(VI) in situ to UO_2_ nanoparticles with a size of 2–5 nm. In the electrocatalytic CO_2_ reduction reaction, with a 2.5 wt% catalyst loading, the minimal onset potential reached −0.77 V (vs. RHE). At −1.27 V polarization, current density attained 15 mA cm^−2^, while the application of −1.37 V yielded 85.5% formate selectivity with ≥91.4% Faradaic efficiency for total C1 products, demonstrating excellent catalytic performance (Figure 2e,f).

Bi-based catalysts have shown excellent catalytic activity and selectivity in the electrocatalytic reduction of CO_2_ to synthesize formate [52]. Addressing the need for efficient formate production, Guo et al. [53] doped trace amounts of Bi (atomic concentration: 740 ppm) into liquid metal Ga. Bi achieved surface enrichment exceeding 400 times in the liquid state (atomic concentration: 30%) with no atomic aggregation. This process generated a catalytically active liquid bismuth interface on liquid Ga at ambient conditions, exhibiting remarkable efficacy for CO_2_ electroreduction. Over an 80 h operational period, the Faradaic efficiency for CO_2_ conversion to formate reached as high as 98%. Ga-based liquid metal catalysts can also convert CO_2_ into CO. Zhang et al. [54] synthesized heteroatom-doped Ga-based single-atom catalysts (Ga SACs) through the polymer pyrolysis strategy, and their dynamic coordination structure significantly improved the CO_2_ electroreduction performance. At a potential of −0.3 V (vs. RHE), the Faradaic efficiency of CO exceeded 92%.

#### 3.1.2. Electrocatalytic Ammonia Synthesis

In the fields of electrocatalytic ammonia synthesis and nitrate reduction to ammonia, Ga-based liquid metals exhibit remarkable advantages due to their unique physical and chemical properties. On the one hand, doping with other atoms can optimize the adsorption of reactants and the conversion pathways of intermediates, thereby enhancing the activity and selectivity of the reaction. On the other hand, their liquid nature endows the catalyst surface with dynamic repair capabilities, effectively addressing the deactivation problem during the catalytic process. This provides an innovative approach for efficient and low-cost electrocatalytic conversion.

**Figure 3:**
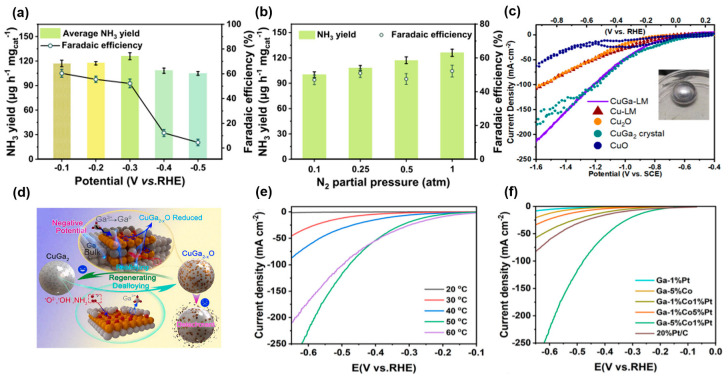
(**a**) Ammonia yield and corresponding Faradaic efficiency of Ru_0.06_/LM@C catalyst in high-purity N_2_ atmosphere [55]. (**b**) Ammonia yield and Faradaic efficiency at different N_2_ partial pressures [55]. (**c**) Cyclic voltammetry of different crystal electrodes was performed in 0.5 M Na_2_SO_4_ + 0.1 M NaNO_3_, and the inset is a photograph of CuGa–LM [31]. (**d**) Schematic diagram of the deactivation-regeneration mechanism [31]. (**e**) Linear sweep voltammetry (LSV) curves of Ga-5wt%Co1wt%Pt (weight ratio g g^−1^) in natural seawater for the electrocatalytic hydrogen evolution reaction (HER) at 20–60 °C [59]. (**f**) Compare the LSV curves of different catalysts at 50 °C LSV [59].

Currently, the nitrogen reduction reaction is conducted in N_2_-saturated environments using high-purity N_2_ as feedstock, which incurs substantial costs. Wei et al. [55] reported a strategy employing 4d metal (Ru)-doped liquid Ga (Ru_0.06_/LM@C) for efficient electrochemical ammonia synthesis across a broad N_2_ concentration range. The experiments were performed in 0.1 mol/L KOH electrolyte, where the catalyst demonstrated superior performance across a broad range of N_2_ concentrations. Under high-purity N_2_ feed, the NH_3_ production rate reached a maximum of 126.0 μg h^−1^ mg_cat_^−1^ at −0.3 V vs. RHE, with a maximum Faradaic efficiency of 60.4% at −0.1 V vs. RHE (Figure 3a), and stable operation for 100 h was achieved in an anion exchange membrane electrolyzer. Twelve cycling tests were conducted, showing no significant decline in catalyst activity after each test. Even at lower N_2_ concentrations, relatively good ammonia synthesis performance was maintained, with Faradaic efficiency consistently exceeding 47% and production rates greater than 100 μg h^−1^ mg_cat_^−1^ (Figure 3b). The fluidity of liquid Ga promotes dynamic surface reconstruction and the continuous exposure of active sites, while Ru doping optimizes the electronic structure, enhancing N_2_ adsorption and intermediate conversion, with the carbon coating simultaneously preventing nanoparticle agglomeration and oxidation. Mechanistic studies revealed that Ru doping enhances the adsorption of N_2_ and nitrogen-containing intermediates through electronic structure modulation (Ga→Ru charge transfer confirmed by XPS/DFT); concurrently, the dynamic surface reconstruction capability of liquid Ga increases the density of active sites and promotes product desorption. These two factors synergistically optimize the N_2_ mass transfer pathway, increasing the coverage of the key *NNH intermediate, thereby broadening the adaptability to varying N_2_ concentrations.

The electrochemical conversion of nitrate to ammonia eliminates hydrogen usage and can potentially integrate electricity from sustainable sources like wind and solar energy. This approach further enables decentralized ammonia production at ambient temperature. Consequently, electrocatalytic nitrate reduction for ammonia synthesis has garnered significant attention as a complementary production method [56]. Jessica et al. [57] employed liquid Galinstan alloy as an electrocatalyst for the nitrate reduction to ammonia, achieving 100% Faradaic efficiency and an ammonia production rate of 2335 µg h^−1^ cm^−2^ at −0.74 V using either 100 mM HNO_3_ or 0.025 M H_2_SO_4_ + 0.025 M NaNO_2_ as the electrolyte, while maintaining 74% Faradaic efficiency after 21 h of continuous operation. The InSn alloy within Galinstan was identified as the primary source of catalytic activity. DFT calculations revealed that the In_3_Sn nanoparticles formed during electrolysis render the reaction free energy downhill throughout the pathway while suppressing the hydrogen evolution reaction; its dynamic surface reconstruction and high density of active sites collectively contribute to long-term stability, and the rapid desorption of NH_3_ prevents active site blocking. Deactivation primarily stemmed from slight surface oxidation and the dynamic reconstruction of the InSn active zones, but the fluidity of the liquid metal enables self-repair via Ga migration and the redistribution of InSn nanoparticles, resulting in significantly lower performance decay compared to solid-state catalysts. Cao et al. [31] utilized the fluidity and alloying capability of liquid Ga to fabricate a flowable and regenerable CuGa_2_–liquid metal electrode for nitrate electrolysis, employing 0.5 M Na_2_SO_4_ + 0.1 M NaNO_3_ as the catholyte solution with testing conducted at 35 °C. Electrochemical tests demonstrated its superior activity and selectivity in the nitrate reduction reaction. As shown in Figure 3c, cyclic voltammetry (CV) analysis revealed that CuGa–LM achieved substantially higher current densities than conventional Cu-based catalysts during nitrate reduction. This enhanced electrochemical activity demonstrates CuGa–LM’s superior catalytic performance for nitrate reduction conversion, peaking at 71.47 mA cm^−2^ under optimal conditions (−1.1 V vs. SCE/−0.43 V vs. RHE) where Faradaic efficiency reached its maximum. However, catalyst detachment due to interfacial evolution occurred. Their study revealed deactivation mechanisms and proposed a regeneration strategy (Figure 3d). The application of a −4 V voltage for 6 s triggered rapid convection in the Ga matrix and realloying of CuGa_2_, enabling repair within seconds and prolonging the catalyst’s lifespan by approximately 1500% compared to the control group. Dynamic surface reconstruction enhances mass transfer via the Marangoni effect in the flowing Ga matrix (mass transfer coefficient: 0.0127 m/s), while the dealloying–realloying cycle of nanoparticles maintains the dispersity and chemical stability of active sites. This synergy significantly enhances the long-term activity and stability of the catalyst.

#### 3.1.3. Electrocatalytic Hydrogen Production

Hydrogen production through water or seawater electrolysis represents a critical step toward a sustainable hydrogen economy [58]. However, conventional alkaline and proton exchange membrane (PEM) electrolysis technologies require high-purity water as feedstock. Due to the necessity for multi-stage pretreatment of natural water sources to meet electrolysis requirements, this process significantly increases hydrogen production costs. Furthermore, large-scale hydrogen production would compete with potable water and agricultural water resources. While the direct utilization of seawater as an electrolysis feedstock eliminates freshwater dependency and reduces energy consumption, this strategy faces challenges including inefficient hydrogen evolution reaction (HER) kinetics and sluggish reaction rates under near-neutral conditions, concurrently accompanied by stability degradation caused by Mg(OH)_2_ and Ca(OH)_2_ precipitate formation at the cathode.

To address these challenges in direct seawater electrolysis, Li et al. [59] reported a dynamic diatomic synergistic catalyst based on liquid metal Ga (Ga–CoPt) for direct seawater electroreduction. The study revealed that liquid Ga effectively disperses Co and Pt atoms, forming a dynamic interface that significantly enhances catalytic activity and stability. Using natural seawater (pH = 8.3) as the electrolyte, Figure 3e presents the LSV curves of Ga–CoPt at various temperatures. At 50 °C, the liquid Ga–CoPt exhibited exceptional performance, demonstrating an overpotential of merely 249 mV at a current density of 10 mA cm^−2^ and a Tafel slope of 145 mV dec^−1^. To probe the influence of Co and Pt on the reaction, Figure 3f shows that increasing the Pt content did not significantly improve HER activity, whereas decreasing the Co content markedly reduced HER activity, indicating that Co plays a pivotal role in the seawater electroreduction process over the Ga–CoPt catalyst. Concurrently, the addition of a small amount of Pt substantially enhanced the catalytic activity, suggesting a synergistic effect between Co and Pt atoms. Catalyst deactivation primarily stems from the physical blockage of active sites by Mg(OH)_2_ and Ca(OH)_2_ precipitates formed during seawater electrolysis. However, the fluidity of liquid Ga enables dynamic migration of Co/Pt atoms to the surface, achieving self-replenishment of active sites. In solid-state Ga–CoPt, Co/Pt atoms form clusters, whereas in the liquid state, the atomic spacing expands to 4.3–6.5 Å (confirmed by AMD simulations), exhibiting long-range disordered atomic-level dispersion. This reconstruction increases the exposure probability of Co/Pt, elevating the electrochemical active surface area from 0.108 mF cm^−2^ in the solid state to 7.95 mF cm^−2^ in the liquid state. In situ Raman spectroscopy and theoretical calculations indicate that in the liquid phase, Co atoms adsorb and dissociate H_2_O to generate H_3_O^+^, while Pt atoms adsorb H* and synergistically produce H_2_ via the Heyrovsky step (ΔG_H_* = −0.093 eV). This avoids the non-uniform active sites and elevated OH* desorption energy barrier (ΔG = +0.27 eV) observed in solid-state catalysts due to Co/Pt clustering.

#### 3.1.4. Electrocatalytic Oxidation of Alcohols

The commercialization of direct alcohol fuel cells (such as methanol and ethanol fuel cells) faces a core challenge in the efficient electrocatalytic oxidation of alcohol fuels. Overcoming this challenge hinges critically on the development of anode catalysts that simultaneously exhibit high activity, high stability, and low cost. Although Pt-based catalysts are widely employed, they remain constrained by limitations including insufficient activity, susceptibility to poisoning by reaction intermediates, and high cost. Consequently, Pt-based catalyst systems utilizing liquid Ga metal as a matrix (e.g., Ga–Pt) demonstrate significantly enhanced catalytic performance in alcohol oxidation reactions, offering a promising solution for this field.

Rahim et al. [33] reported a study on a low temperature liquid Pt catalyst based on Ga. The Ga–Pt catalyst exhibited superior catalytic activity for electrochemical methanol oxidation, achieving a mass activity of ~2.8 × 10^7^ mA mg^−1^ Pt, which was three orders of magnitude higher than that of existing Pt catalysts. No significant deactivation was observed during a 1 h chronoamperometric response test. The excellent catalytic performance is attributed to the dynamic dispersion of Pt atoms within the liquid Ga matrix and the alteration of their electronic structure. DFT calculations identified possible reaction pathways on the Ga–Pt surface and revealed through Gibbs free energy diagrams that the energy barriers for key steps, such as methanol dissociation (*CH_3_OH → *CH_3_O + H), were significantly lower on the G–Pt surface compared to the pure Ga surface. This confirmed that Pt doping effectively optimized the reaction kinetics. However, the mechanism by which the liquid catalyst enhances activity remained unexplored. Lambie et al. [60] systematically analyzed the dynamic behavior of the Ga–Pt catalyst in both isolated states and under adsorbate influence using Ab initio molecular dynamics (AIMD) simulations. The role of Pt extends beyond acting solely as a direct catalytic active site; its key function lies in dynamically activating neighboring Ga atoms through electronic and geometric effects. The migration of Pt atoms within the liquid Ga matrix induces dynamic adjustments in the local electronic structure of Ga sites, forming Pt-induced Ga active sites. These activated Ga sites exhibit significantly reduced adsorption energies for key reaction intermediates, such as *CH_3_O.

**Figure 4:**
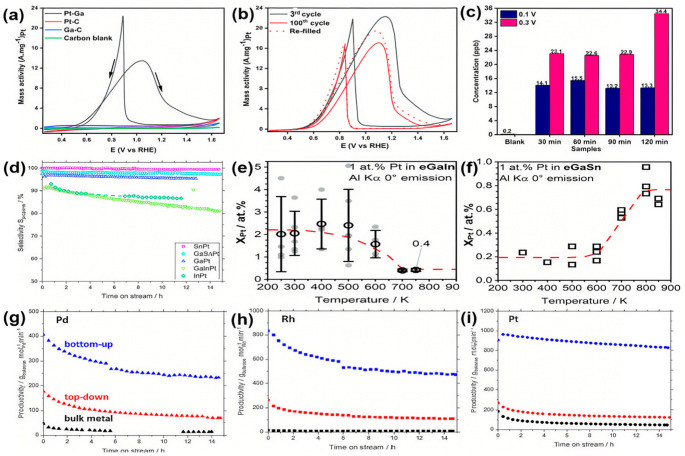
(**a**) EOR-specific activities of different catalysts in 0.03 M KOH with 4 M ethanol solution [34]. (**b**) Comparison of EOR performance of Pt–Ga between 3rd and 200th cycles [34]. (**c**) ICP-MS analysis of electrolytes cycled at 0.1 and 0.3 V vs. Ag/AgCl for 2 h [34]. (**d**) The propylene selectivity of binary and ternary catalyst systems was compared in the propane dehydrogenation reaction at 823 K and 1.2 bar [61]. (**e**) Temperature-dependent Pt content in the near-surface region for GaInPt [61]. (**f**) Temperature-dependent Pt content in the near-surface region for GaSnPt [61]. Comparison of catalytic productivity in isobutane dehydrogenation at 723 K and 1.2 bar between a conventional bulk metal catalyst and Ga–SCALMS systems. Ga–SCALMS catalysts were synthesized via ultrasonication and a chemical route. Relative performance is shown for each noble metal component: (**g**) Pd, (**h**) Rh, and (**i**) Pt [40].

Advancing direct ethanol fuel cells requires high-performance electrocatalysts with both efficiency and operational durability. Nazir et al. [34] reported Pt–Ga liquid metal nanodroplets as superior electrocatalysts for ethanol oxidation. In alkaline electrolyte, the catalyst achieved a mass activity of 13.47 A mg^−1^ Pt, which was over 14 times higher than commercial Pt/C catalysts. Figure 4a further revealed an onset potential of 0.42 V (vs. RHE), substantially lower than literature benchmarks, confirming exceptional ethanol oxidation reaction performance. It demonstrated good stability in cyclic voltammetry tests (Figure 4b), maintaining high activity after 100 cycles and recovering 87% of its initial activity upon electrolyte replacement. After cycling for 2 h at 1.06 V vs. RHE, Ga concentration in the electrolyte was 13.3 ppb, remaining nearly constant from 30 to 120 min, indicating electrode stability under low-voltage conditions (Figure 4c). Catalyst deactivation primarily originates from Ga dissolution at high potentials (>1.16 V vs. RHE), reaching a concentration of 34.4 ppb in the electrolyte at 1.26 V, which can be mitigated through potential control. Dynamic surface reconstruction achieves atomic-level dispersion of Pt atoms via the liquid Ga matrix, suppressing agglomeration; concurrently, the surface self-forming Ga_2_O_3_ layer stabilizes reaction intermediates and promotes desorption, preventing poisoning. This approach represents a new pathway for high performance ethanol fuel cells.

The high activity and selectivity of liquid Pt catalysts for alcohol oxidation originate from unique electronic and geometric structural alterations at the interface. XPS and Bader charge analyses reveal that Pt atoms acquire a negative charge due to electron donation from Ga. This lowers the d-band center and weakens the adsorption strength of reaction intermediates, thereby suppressing over-oxidation. AIMD simulations demonstrate that Pt atoms are dynamically dispersed within the liquid Ga matrix (diffusion coefficient ≈ 0.23 × 10^−5^ cm^2^ s^−1^), preventing the formation of Pt–Pt bonded clusters. Lateral displacements continuously expose regions of high reactant concentration. Furthermore, the synergistic interaction between surface Ga oxide (Ga_2_O_3_) and platinum stabilizes key intermediates, reducing the energy barrier for the methanol oxidation pathway by 0.37 eV. In ethanol oxidation, Ga oxide acts as a proton acceptor, promoting the formation of acetaldehyde/acetic acid instead of C–C bond cleavage products. This dynamic interface combined with electronic modulation collectively enables highly selective partial alcohol oxidation.

### 3.2. Application of Ga-Based Liquid Metals in Thermal Catalysis

Ga-based liquid metal catalysts exhibit remarkable coking resistance in thermocatalytic reactions due to their dynamic surface properties and low surface energy. This characteristic effectively prevents the deposition and agglomeration of carbonaceous intermediates on the catalyst surface, avoiding deactivation through active site coverage, thereby maintaining high reaction efficiency of the catalytic system over prolonged periods. In industrial alkane dehydrogenation processes, catalyst deactivation by coking constitutes a primary challenge. Owing to their liquid nature, these catalysts demonstrate significant application potential for olefin production via hydrocarbon dehydrogenation. Furthermore, they hold substantial promise for other thermocatalytic reactions including alkane dehydrogenation reaction, selective hydrogenation, CO_2_ reduction, ammonia synthesis, and thermocatalytic plastic degradation.

#### 3.2.1. Thermocatalytic Alkane Dehydrogenation

Short-chain olefins, particularly propylene and butylene, are among the most important fundamental chemicals due to their role as fundamental chemical feedstocks and intermediates for a wide range of products [62]. Ga-based liquid metal catalysts, with unique liquid-phase properties, high activity, and excellent selectivity, show great potential in hydrocarbon dehydrogenation reactions. Adjusting metal components, adding third elements, optimizing supports, and improving preparation methods can enhance their performance, achieving efficient, stable olefin synthesis with high selectivity. Their dynamic surface reconstruction and alloying behavior also enable superior performance in complex reaction conditions.

Taccardi et al. [41] reported a supported liquid metal catalyst (Ga–Pd SCALMS), prepared by dissolving Pd in low melting point Ga to form a liquid metal phase and supporting the Pd–Ga liquid alloy on a porous glass support. Butane dehydrogenation (BDH) to butene was performed at 445 °C and 11 bar. When feeding He:C_4_H_10_ = 10:1, the catalyst with a Ga/Pd molar ratio = 52 exhibited the highest activity (TOF = 39 h^−1^) and 85% butene selectivity, with almost no activity decay after 20 h of continuous operation. Under pure butane feed for 100 h, only minor deactivation occurred (deactivation rate < 5%), whereas an industrial Pt/Al_2_O_3_ catalyst deactivated by 40% within 20 h. Deactivation in industrial catalysts primarily stems from coke deposition, but the high fluidity of liquid Ga enables the dynamic migration of Pd atoms to the interface, preventing sintering; in situ XRD confirmed no crystalline phase separation at 550 °C. Moreover, Pd uniformly dissolves in the liquid Ga matrix with a surface Pd atomic concentration < 0.3%, eliminating coke formation caused by adjacent active sites in conventional catalysts. During operation, a thin Ga_2_O_3_ layer forms on the surface (XPS), and its dynamic reconstruction removes coke precursors, maintaining active site exposure. Raman et al. [42] prepared a Ga–Rh SCALMS catalyst for propane dehydrogenation by partially electrochemically replacing Rh into a Ga/AlO_x_ material. When the Ga/Rh ratio exceeded 80, the catalyst’s activity increased dramatically while maintaining high propylene selectivity. At a Ga/Rh ratio of 125, the carbon deposition on the catalyst at 450 °C was 0.3–0.6 wt%, significantly lower than that observed on Rh/Al_2_O_3_ (>1 wt%). Catalyst deactivation resulted from initial carbon deposition forming highly reactive “soft coke” on the liquid Ga surface, which subsequently migrated to the Al_2_O_3_ support to form “hard coke”, thereby blocking the pore channels. However, after treatment under 21% O_2_/He at 500 °C for 2 h (GHSV 30,000 mL·g^−1^·h^−1^), both the soft coke (igniting from 150 °C) and the hard coke (oxidized between 350 and 500 °C) were completely oxidized to CO_2_. The catalyst remained recoverable after five cycles, although its activity gradually declined due to the irreversible pore blocking of the support. Raman et al. [44] investigated the propane dehydrogenation performance of Ga–Pt liquid metal solutions (Ga-Pt SCALMS) supported on porous carrier materials within the high-temperature range of 500 to 600 °C. They systematically studied the effects of three carriers (SiO_2_, Al_2_O_3_, and SiC) on PDH activity, selectivity, and coking behavior. The catalyst using SiC as the support demonstrated the best overall performance, achieving both high activity (cumulative yield of 800 g_propene_ g_Pt_^−1^ h^−1^) and moderate stability at high temperature (550 °C).

To investigate the influence of a third component on catalytic performance, Moritz et al. [61] introduced In or Sn into the Ga–Pt binary system to construct GaInPt and GaSnPt ternary liquid alloys. Catalytic performance tests (Figure 4d) revealed that GaPt, GaSnPt, and SnPt systems all exhibited excellent propene selectivity (>95%). TPXPS characterization demonstrated that the third component significantly altered the surface composition and concentration gradient. Despite both GaInPt and GaSnPt alloys having the same nominal Pt content of 1 at%, their Pt surface distribution behavior exhibited fundamental differences. Below 700 K, the GaInPt system displayed Pt enrichment in the near-surface region (concentration significantly higher than GaSnPt). When the temperature was raised above 700 K, system homogenization stabilized the Pt surface concentration at 0.4 at% (representing a 60% reduction compared to the nominal bulk concentration) (Figure 4e). In contrast, below 800 K, the GaSnPt system exhibited Pt depletion at the surface (Figure 4f), primarily attributed to the precipitation of Pt-rich intermetallic phases within the bulk. After the temperature increased above 800 K, Pt became uniformly dispersed, stabilizing the surface concentration at 0.8 at% (a 20% reduction compared to the nominal bulk concentration). These differences indicate that In induces the preferential precipitation of Pt-rich phases near the surface, resulting in significant Pt surface loss due to migration into the bulk upon liquefaction at elevated temperatures; conversely, Sn promotes Pt segregation within the bulk, leading to higher Pt surface retention after high-temperature homogenization. Consequently, the GaSnPt system demonstrates superior catalytic performance.

To compare the influence of synthesis methods on the catalytic performance of Ga-based SCALMS catalysts for isobutane dehydrogenation, Raman et al. [40] systematically compared the effects of two synthesis methods (bottom-up approach and top-down approach) on catalyst structure, activity, and stability. The top-down approach using ultrasonication was denoted as “td”, while the bottom-up approach via chemical deposition was denoted as “bu”. Their study found that SCALMS catalysts (GaPd, GaRh, and GaPt) outperformed monometallic catalysts in both activity and stability. Under reaction conditions at 723 K, the SCALMS catalysts prepared via the bottom-up method exhibited higher catalytic activity. The initial butene yields for GaPd_bu/Al_2_O_3_, GaRh_bu/Al_2_O_3_, and GaPt_bu/Al_2_O_3_ catalysts prepared by the bottom-up approach were 407 g_butenes_ mol_M_^−1^ min^−1^, 832 g_butenes_ mol_M_^−1^ min^−1^, and 963 g_butenes_ mol_M_^−1^ min^−1^, respectively, over twice that of catalysts prepared by the top-down method (as shown in Figure 4g–i). Furthermore, the bottom-up synthesized catalysts demonstrated lower deactivation rates during the reaction, with the GaPt_bu catalyst notably maintaining nearly 85% of its initial activity after 14 h. In contrast, catalysts prepared by the top-down method, due to their larger liquid metal droplets primarily located on the outer surface of the support, resulted in a lower utilization of active sites.

The use of supported catalysts not only reduces the required amount of metal but also mitigates numerous corrosion issues in reactor design, as contact between the liquid metal and the reactor walls can be minimized. In SCALMS studies for alkane dehydrogenation, fixed-bed continuous flow reactors are widely adopted due to their compatibility with static supported liquid metal catalysts. SCALMS consist of liquid metal alloy droplets anchored within a porous rigid support (such as SiO_2_, Al_2_O_3_, SiC, or porous glass). The droplet size (0.1–10 μm) is well suited to the static packing mode of fixed beds, and the dynamic nature of the liquid interface at high temperatures (445–600 °C) eliminates the need for catalyst flow. The reactor, typically a quartz tube, is packed with catalyst and placed within a split-tube furnace for precise temperature control. Diluted alkane is fed at a defined space velocity to achieve continuous flow. Products are analyzed in real-time using online gas chromatography, enabling validation of the long-term coke resistance of SCALMS under industrially relevant conditions.

Beyond propane dehydrogenation for propylene production, the selective cracking of long-chain hydrocarbons (such as decane and biomass-derived rapeseed oil) represents another significant pathway for synthesizing propylene. Junma Tang et al. [7] reported the use of a liquid GaSn_0.029_Ni_0.0023_ alloy catalyst for the selective synthesis of propylene. Combining computational simulations with experimental validation, they proposed the following reaction mechanism: Interfacial Sn atoms protrude outward, while subsurface Ni atoms undergo dynamic rearrangement to precisely match decane molecules, synergistically promoting propylene formation. Under conditions of approximately 150 °C, this catalyst achieved the synthesis of high-purity propylene from decane with a selectivity of approximately 90.5%. It was also successfully applied to the conversion of rapeseed oil, achieving a propylene selectivity as high as approximately 94.5%. In the rapeseed oil conversion reaction, the liquid GaSn_0.025_Ni_0.025_ catalyst demonstrated excellent long-term stability during continuous operation for 720 h (30 days), with the propylene yield decreasing from an initial 2.0 × 10^−3^ mol min^−1^ to 1.7 × 10^−7^ mol min^−1^, attenuating by only 15%.

#### 3.2.2. Selective Hydrogenation

Selective hydrogenation refers to the process in which, when a reactant contains two reducible functional groups, the targeted functional group is hydrogenated while the other remains unaffected. This process finds wide application in industries such as petrochemicals and fine chemicals [63]. The key to selective hydrogenation lies in fabricating catalysts with high efficiency and selectivity [64]. In the development of highly efficient hydrogenation catalysts, achieving precise control over the geometric arrangement and electronic structure of active sites to balance activity and selectivity while inhibiting deactivation represents a core challenge. Ga, with its unique physicochemical properties, offers a novel perspective for addressing this challenge. In recent years, utilizing Ga as a key component, a series of novel catalytic systems have been developed. These systems are constructed either by forming intermetallic compounds with transition metals (e.g., Pd, Ni, Pt), by serving as a support for loading active metals, or even by Ga itself forming active hydride species. These systems demonstrate outstanding performance.

Armbrüster et al. [65] reported a series of Pd–Ga intermetallic compound catalysts (including Pd_3_Ga_7_, PdGa, and Pd_2_Ga) for the selective hydrogenation of acetylene. Under conditions of 180 °C and 0.1 MPa, the Ni_5_Ga/SiO_2_ catalyst (Ni/Ga = 5) exhibited an optimal performance, maintaining 100% acetylene conversion and achieving 81% ethylene selectivity over 120 h. Their performance originates from the strong interaction formed between Pd and Ga: On one hand, the ordered crystal structure of the intermetallic compounds achieves the geometric isolation of Pd active sites; on the other hand, the Pd–Ga bond modulates the electronic structure of Pd, reducing the density of states (DOS) near the Fermi level. These two effects synergistically suppress the occurrence of side reactions. Catalyst deactivation is primarily caused by coke deposition blocking pores and the formation of NiC_x_, which can be regenerated via high-temperature coke combustion at 600 °C followed by H_2_ reduction. Li et al. [66] synthesized Pd–Ga catalysts supported on three polymorphs of Ga_2_O_3_ (α, β, γ) via controlled coprecipitation and thoroughly investigated the formation of Pd–Ga intermetallic compounds and their selectivity and stability in the selective hydrogenation of acetylene. During hydrogen reduction, the catalysts supported on α- and β-Ga_2_O_3_ formed the Pd_2_Ga compound at 250 °C and 310 °C, respectively, which further enriched in Ga to form Pd_5_Ga_3_ at higher temperatures. Pd_2_Ga maintained a stable ethylene selectivity of approximately 75% in acetylene hydrogenation, higher than that of monometallic Pd catalysts, whereas the selectivity of Pd_5_Ga_3_ could reach 80%. Wang et al. [67] compared the catalytic performance in selective acetylene hydrogenation of Ni_x_Ga/SiO_2_ catalysts with different Ni/Ga atomic ratios (x = 10~2) and Ni/SiO_2_ catalysts. The results indicated that Ni_x_Ga/SiO_2_ catalysts exhibited higher ethylene selectivity than Ni/SiO_2_ catalysts. This behavior stems from Ni–Ga alloy and Ni_3_Ga intermetallic phase formation. Electron donation from Ga to Ni weakens ethylene binding on active sites while reducing coverage, consequently suppressing over-hydrogenation, C–C cleavage, and polymerization pathways.

**Figure 5:**
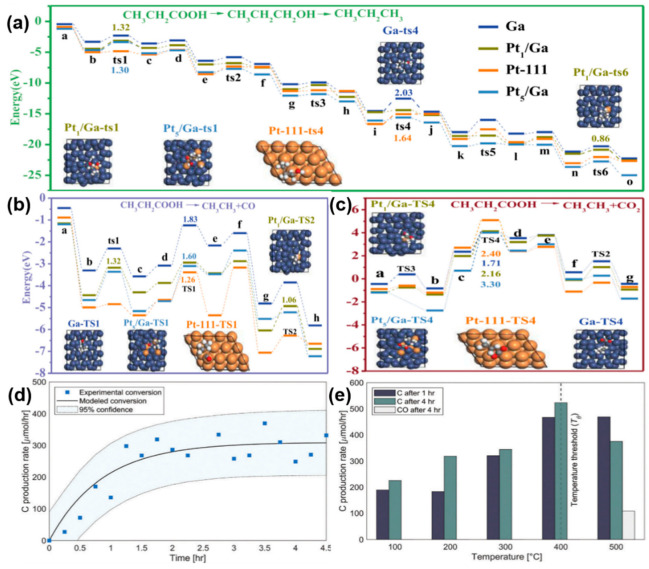
(**a**–**c**) Fatty acid hydrogenation conversion pathway on Ga, Pt_1_/Ga, Pt_5_/Ga, and Pt-111 catalyst surfaces (In Figures a-c, a-o, a-h, and a-g represents the energy state nodes corresponding to the elementary reaction steps under the action of different catalysts, representing the successive energy change stages experienced during the reaction process.) [32]. (**d**) The carbon production rate when using EGaIn alloy to catalyze the conversion of carbon dioxide into carbon under continuous CO_2_ flow in a bubbling column reactor at 200 °C and ambient pressure [69]. (**e**) Arrhenius analysis of temperature-dependent logarithmic rate constants enables experimental determination of activation energies [69].

Long et al. [32] successfully prepared atomically dispersed Pt_x_/Ga catalysts via ultrasonic emulsification and impregnation methods, which exhibited excellent hydrogenation performance without requiring a conventional reduction process. Pt atoms were uniformly embedded as isolated atoms within the liquid Ga matrix, effectively avoiding aggregation issues. The 0.5 wt% Pt_x_/Ga catalyst achieved 100% conversion of stearic acid and 98.7% selectivity towards octadecane in the hydrogenation reaction and maintained exceptional stability even after 19 cycles. To improve the efficiency of DFT calculations, CH_3_CH_2_COOH, a low-carbon-chain acid sharing the same functional group as stearic acid, was selected. Figure 5a–c provides the energy profiles for alkane formation from the acid, including three distinct pathways. The charge transfer from Ga to Pt optimizes the d-band center to strengthen the hydrogenation activity while weakening CO adsorption capacity, effectively preventing catalyst poisoning and aggregation. Concurrently, the catalyst’s strong adsorption capacity for aldehyde and alcohol intermediates facilitated stearic acid hydrogenation through hydrodehydration, enhancing carbon efficiency substantially. Manyuan et al. [68] introduced a novel method for preparing Ga hydride (Ga–H)-modified nanoparticles (Ga–H@LM NPs) by ultrasonically activating liquid metal in water and confirmed their role as highly efficient hydrogenation active sites. When applied to the catalytic hydrogenation reaction of 4-nitrophenol (4-NP), Ga–H@LM NPs alone, without any additional hydride reducing agents, converted 4-NP to 4-aminophenol (4-AP) within 5 min. This reaction rate significantly outperforms that of conventional catalysts.

#### 3.2.3. Thermocatalytic CO_2_ Reduction

Despite the significant potential of thermocatalytic CO_2_ reduction in carbon dioxide mitigation and resource utilization, the process requires high temperatures, leading to increased energy consumption. Consequently, reducing energy demand and enhancing reaction efficiency constitutes a critical research direction. Ga-based materials exhibit remarkable potential for breakthrough applications in CO_2_ catalytic conversion. Their unique low melting point characteristics and tunable electronic structure can significantly reduce reaction energy barriers, enabling efficient reduction processes at low temperatures.

Studt et al. [70] employed Ni–Ga catalysts for the reduction of CO_2_ to methanol. At temperatures above 220 °C, the methanol yield was significantly higher than that of the conventional Cu/ZnO/Al_2_O_3_ catalyst, while simultaneously substantially reducing the proportion of CO generated via the reverse water–gas shift reaction. Karma et al. [69] reported the direct decomposition of CO_2_ into solid carbon at ambient temperature using the EGaIn liquid metal alloy as the catalyst. Exploiting the alloy’s low melting point to promote low-temperature CO_2_ reduction, the carbon production rate increased over time at 200 °C, reaching a stable rate of 319 μmol h^−1^ after 150 min (as shown in Figure 5d). The carbon productivity of EGaIn increased with rising temperature (Figure 5e). The activation energy for CO_2_ decomposition over EGaIn, determined via the Arrhenius model, was 8.39 kJ/mol, indicating low energy requirements enabling the reaction to proceed at relatively low temperatures. During extended testing, EGaIn maintained stable activity after 24 h of continuous operation at 200 °C. The catalyst achieved CO_2_ activation even at room temperature, generating solid carbon without requiring auxiliary reducing agents such as hydrogen. No signs of coking-induced deactivation were observed in the liquid metal during the reaction, and the produced carbon accumulated on top of the liquid metal, enabling facile collection.

#### 3.2.4. Thermocatalytic Ammonia Synthesis

Ammonia serves not only as a core raw material for nitrogen fertilizer production, playing an indispensable role in agricultural production, but also finds widespread application across multiple industrial sectors, making it a fundamental chemical feedstock [71]. While ammonia boasts extensive uses and high demand, its current global production primarily relies on high energy consumption and resource-intensive Haber–Bosch process, which is highly consuming of both energy and resources [72].

In order to reduce the reaction conditions for ammonia synthesis, Karma et al. [30] reported the conversion of nitrogen and hydrogen to ammonia using the liquid Cu–Ga alloy under mild conditions (400 °C, 4 bar). By leveraging the synergistic effect between Cu and Ga, along with the tunable electronic and geometric structures of the liquid metal alloy, activity far exceeding that obtained with single-component metals was achieved. Without requiring high pressure, an ammonia production rate of approximately 9500 µmol g^−1^ h^−1^ was observed on the Cu–Ga alloy containing 2 wt% Cu at 400 °C and 4 bar. This breakthrough overcomes the energy consumption limitations of the conventional Haber–Bosch process. Under identical reaction conditions, the Cu–Ga catalyst exhibited an ammonia production rate over five times higher than that of the commercial Cs–Ru/MgO catalyst (2.5 wt% Ru). This enhanced performance was attributed to the apparent activation energy for ammonia production over the Cu–Ga catalyst being 25.2 kJ mol^−1^, substantially lower than that of Ru-based catalysts.

#### 3.2.5. Thermocatalytic Plastic Degradation

Polyvinyl chloride (PVC) is ubiquitous in daily life. However, it is non-recyclable and decomposes above 250 °C to produce toxic HCl and chlorinated hydrocarbons. Conventional PVC conversion methods include pyrolysis and catalytic conversion, but these typically require high temperatures and struggle to avoid HCl generation. Additionally, traditional solid catalysts exhibit a limited contact area with PVC, compromising conversion efficiency. Felipe et al. [73] proposed a catalytic route using liquid metal Ga as a catalyst to dechlorinate PVC at 200 °C (~90% Cl removal), producing gaseous H_2_ (with negligible corrosive HCl generated) and carbon materials. PVC powder was mixed with the Ga catalyst and reacted at 200 °C for 1 h. Gas phase composition was characterized using coupled gas chromatography–mass spectrometry (GC–MS), and solid products were analyzed by thermogravimetric analysis (TGA) and XPS. During the Ga-catalyzed PVC conversion, gaseous H_2_ was produced, with its yield proportional to the hydrogen content in PVC. Approximately 90% of the chlorine content in PVC was removed, with the Cl fixed within the solid carbon material and readily removable by simple ethyl acetate washing. The carbon material also possessed a more rigid cross-linked structure.

### 3.3. Photochemical Catalysis

The emergence of Ga-based liquid metal catalysts in photocatalysis is primarily driven by their unique liquid state and superior physical properties. These properties offer a promising new platform and novel strategies to address key challenges faced by conventional solid photocatalysts, such as severe carrier recombination and limited active sites. Ga itself is widely used in optical materials; its high electron mobility and low work function render it an efficient electron donor/acceptor medium. This characteristic is crucial for photocatalytic processes, as it effectively promotes the spatial separation of photogenerated electron–hole pairs and enables the regional enrichment of photogenerated electrons. Leveraging these advantages, researchers are actively developing advanced Ga-based liquid metal systems with higher efficiency, broader spectral response ranges, and even novel photocatalytic functionalities, aiming to advance the practical applications of photocatalysis in energy, environment, and synthetic chemistry. Currently, such catalysts have demonstrated promise in applications like photocatalytic organic decomposition and water splitting.

#### 3.3.1. Photocatalytic Decomposition of Organic Substances

With the rapid development of society, problems of energy crisis and environmental pollution have become increasingly prominent. Against this backdrop, photocatalytic technology has demonstrated significant application prospects in the fields of energy conversion and environmental protection due to its ability to directly convert solar energy into chemical energy under mild reaction conditions. In particular, the utilization of light-driven metal composite photocatalysts for pollutant degradation has garnered significant interest among researchers, coinciding with rising global attention to environmental remediation.

Among strategies for enhancing photocatalytic performance, loading semiconductor materials onto substrates with special properties represents an effective approach. Zhang et al. [74] significantly improved photocatalytic performance by supporting solvent-thermally synthesized γ-Ga_2_O_3_ nanoparticles (5–10 nm) onto a micro/nano spherical framework formed by the liquid metal Galinstan (LM/MO). Under simulated sunlight, with a Ga_2_O_3_ loading of 1 wt%, the degradation efficiency for Congo red reached 100% h^−1^ (complete degradation within 1 h), substantially higher than that of the unmodified LM/MO framework (47% h^−1^) and pure γ- Ga_2_O_3_ (0.03% h^−1^). Notably, the catalyst exhibited good stability, with less than 2% deactivation after four cycles and no significant loss of activity.

Beyond loading strategies, the inherent chemical activity of liquid metals also provides a unique pathway for constructing novel core–shell structured photocatalysts. Ghasemian et al. [75] reported a self-limiting growth strategy based on EGaIn. On the EGaIn liquid metal surface, the strongly oxidizing MnO_4_^−^ ions oxidize liquid Ga to form a monolayer of hydrated MnO_2_, while Ga dissolves as [Ga(OH)_4_]^−^. Concomitant with ultrasonic fragmentation, EGaIn was dispersed into micro/nanodroplets. The ongoing reaction facilitated the near-complete migration of Ga to the surface, where it was oxidized to GaOOH. As the alloy melting point increased upon Ga depletion, the remaining In solidified into a solid metallic core, while the surface formed a porous shell composed of interlocked stacks of hydrated MnO_2_ and GaOOH nanosheets, ultimately forming the In@MnO_2_/GaOOH photocatalyst. This catalyst also exhibited excellent photocatalytic degradation performance for Congo red dye under simulated sunlight (AM 1.5, 100 mW·cm^−2^), maintaining >92% degradation efficiency with <5% decay after five cycles. The enhanced catalytic activity originates from two key features; on one hand, the core–shell structure generates hydroxyl and superoxide radicals under illumination that synergistically degrade the dye, and on the other hand, the interwoven porous shell of MnO_2_ and GaOOH nanosheets provides high adsorption capacity and efficient mass transfer channels, facilitating contact between pollutants and active sites. Furthermore, Liang et al. [76] also employed EGaIn as a matrix to construct core–shell photocatalysts (Cu/W/Mo/Ni-LM@Ga_2_O_3_) with Cu/W/Mo/Ni-doped Ga_2_O_3_ shells encapsulating a liquid metal core via an ultrasonic blending–in situ oxidation strategy. The liquid metal core spontaneously forms a Ga_2_O_3_ semiconductor shell (5–20 nm thick) in the aqueous phase, generating electron–hole pairs under UV light (365 nm) excitation; the holes oxidize pollutants while electrons transfer to the core; and nanoparticles (e.g., highly dispersed Ni) enhance light absorption via the plasmonic effect and modulate the electronic structure. Among these, the Ni–LM system achieved degradation rates of 92.0% and 79.4% for methylene blue and Congo red, respectively, outperforming the pure liquid metal. However, dynamic surface reconstruction during prolonged catalysis causes the irreversible loss of active components (~8% activity decay for Ni–LM after 65 h), limiting its lifespan. Optimizing metal doping can delay deactivation, but long-term stability still requires balancing surface oxidation kinetics with component regeneration capability.

#### 3.3.2. Photocatalytic Overall Water Splitting

Overall water splitting (OWS) represents a highly attractive strategy aimed at converting solar energy into clean hydrogen fuel. This approach utilizes photocatalysts to directly split water into hydrogen and oxygen under light irradiation, thereby enabling the conversion and storage of solar energy. The currently reported solar-to-hydrogen (STH) energy conversion efficiency can even exceed 1%, surpassing that of natural photosynthesis. This progress offers significant promise and impetus for the large-scale application of photocatalytic water splitting technology in sustainable hydrogen fuel production [77].

At the materials design level, NiTiO_3_ (NTO) is an ideal candidate for photocatalytic OWS due to its broad spectral absorption (UV to IR) and earth abundance. Long Wang et al. [78] significantly enhanced the OWS performance of NTO through Ga doping (Ga-NTO). Acting as n-type doping, Ga introduces shallow acceptor states near the conduction band minimum, increasing the hole concentration by approximately threefold and reducing the exciton binding energy from 36.9 meV to 29.8 meV. This extends the lifetime of photogenerated charges and reduces the charge transfer resistance (to 0.24 that of undoped NTO), effectively suppressing charge recombination. Co-based (4 wt%) and Pt-based (1 wt%) cocatalysts functioned as oxygen evolution reaction and hydrogen evolution reaction promoters, respectively, synergistically accelerating surface reaction kinetics. Under visible light (420 nm, pH = 4.9), the H_2_ and O_2_ evolution rates reached 1.81 μmol h^−1^ and 0.90 μmol h^−1^, strictly adhering to the 2:1 stoichiometric ratio, with an apparent quantum yield (AQY) of 0.18%. The AQY further increased to 0.85% under 365 nm UV light. The system maintained stable activity during a continuous 130 h reaction (cumulative gas production > 339 μmol), with the crystal structure remaining intact post-reaction. Hanggara et al. [79] synthesized highly crystalline monoclinic β-Ga_2_O_3_ nanoparticles (particle size ≈ 40 nm) via a solid-state reaction method, achieving efficient UV-light-driven overall water splitting (OWS). To favorably alter the bulk state of β-Ga_2_O_3_, doping with various metals, including alkaline earth metals and first-row transition metals, was performed. Alkaline earth metal cations generally enhance water splitting activity. In contrast, first-row transition metal cations, with the exception of Zn, reduce the activity. Specifically, doping with Zn significantly increased the water splitting activity.

### 3.4. Other Types of Catalytic Reactions

Owing to their unique physicochemical properties, Ga-based liquid metal catalysts also demonstrate significant application potential in electrochemical energy storage and green energy conversion. Their core advantage lies in their capability to optimize key processes. Within electrochemical energy storage systems (e.g., batteries), the high electrical conductivity and electron-donating behavior of liquid metal effectively reduce the activation energy barrier for reactions at the electrode/electrolyte interface, accelerate the charge transfer kinetics across this interface, and enhance the structural stability of the electrode. In the domain of energy catalytic conversion, liquid metal can activate the cleavage and reformation of chemical bonds (e.g., C–H, C–C bonds) under near-ambient temperature conditions, providing an ideal platform for efficient catalysis driven by low-grade mechanical energy and, thereby, circumventing the substantial energy losses associated with conventional high-temperature and high-pressure processes.

**Figure 6:**
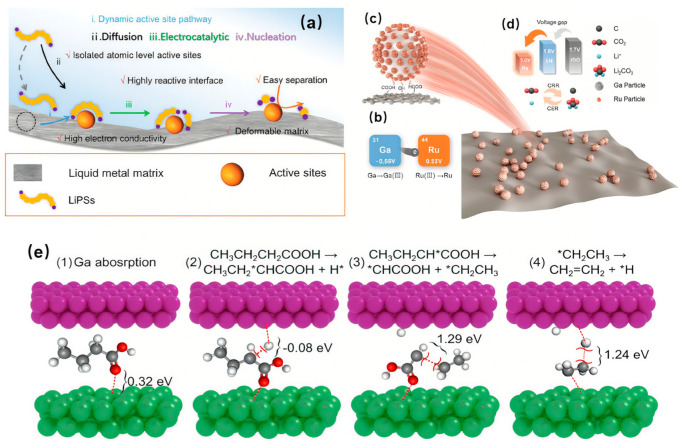
(**a**) A schematic illustration of the catalytic strategies for dynamic liquid metal electrocatalysts [82]. (**b**) A GRR induced by electrode potential difference [84]. (**c**) The core–shell structure of LM@Ru and its attachment to rGO [84]. (**d**) The rGO@LM@Ru cathode with a significantly reduced CRR/CER voltage gap in Li–CO_2_ cell [84]. (**e**) DFT for butyric acid processing were correlated with co-catalyst interfacial reaction mechanism schematics [88].

In the field of electrochemical energy storage, lithium–sulfur (Li–S) batteries are regarded as strong contenders for next-generation energy storage technology due to their high theoretical energy density (2600 Wh kg^−1^) and environmental friendliness [80]. However, their practical application is limited by the lithium polysulfide (LiPS) shuttle effect and sluggish redox kinetics. [81]. Qi et al. [82] introduced EGaSn as a dynamic liquid metal catalyst in their research on promoting LiPSs redox reactions; the strategy for dynamic liquid metal catalysis is depicted in Figure 6a. Their study revealed that this catalyst demonstrated exceptional electrocatalytic performance: The activation energy for the LiPSs reduction process is reduced to 0.56 eV, significantly enhancing interfacial charge transfer efficiency and effectively suppressing the LiPSs shuttle effect. Benefiting from these superior properties, the Li–S pouch cell based on the EGaSn catalyst achieved a specific energy density as high as 307.7 Wh kg^−1^. Furthermore, even under demanding conditions of high sulfur loading (5 mg cm^−2^) and a low electrolyte/sulfur ratio (3.8 µL mg^−1^), the cell still exhibited excellent cycling stability, with an average capacity decay rate of only 0.51% per cycle. Wang et al. [83] reported an ambient-temperature battery system featuring solid lithium anodes paired with Ga–Sn liquid metal cathodes. Through the interface engineering of current collectors and implementation of microstructure-refined cathode droplets, the design achieved exceptional cycling stability with minimal self-discharge. Performance evaluations confirmed specific capacities reaching 409 mAh g^−1^ and energy efficiencies up to 92% in Li||Ga–Sn configurations.

Beyond energy storage, Ga-based liquid metals also demonstrate unique value in the field of CO_2_ capture, utilization, and storage. Li–CO_2_ batteries represent an innovative solution integrating both energy storage and CO_2_ utilization. To address the issue of cathode passivation caused by insulating Li_2_CO_3_ deposits in Li–CO_2_ batteries, Chai et al. [84] proposed a liquid metal-based composite cathode catalyst (rGO@LM@Ru) (as shown in Figure 6b–d) to address the issue of cathode passivation caused by the deposition of insulating Li_2_CO_3_ in Li–CO_2_ batteries. This catalyst features an in situ-generated Ru shell on the surface of liquid metal particles (GaIn_10_) via a galvanic replacement reaction (GRR), forming an LM@Ru core–shell structure anchored onto a reduced graphene oxide (rGO) support. From the perspective of electron-donating properties, the liquid metal optimizes the charge transport pathway. XPS analysis confirmed electron donation from liquid metal to Ru (evidenced by a negative shift in Ru binding energy), significantly reducing electrochemical polarization. Concurrently, hydrogen-bonding interactions form between the oxide layer on the liquid metal surface (Ga_2_O_3_) and the oxygen-containing functional groups (-OH/-COOH) of rGO. This interaction endows the catalyst with uniform loading and structural stability. The synergistic mechanisms described above collectively enable the rGO@LM@Ru cathode to achieve a low voltage gap of 1.0 V, a long cycle life exceeding 500 h, enhanced capacity (reaching 4128 μAh cm^−2^), and improved rate capability. Furthermore, by regulating the deposition morphology of Li_2_CO_3_ (resulting in small-sized, uniformly distributed deposits), the reaction reversibility is significantly enhanced.

Ga-based liquid metals provide an ideal platform for the efficient utilization of low-grade mechanical energy, particularly in the field of green energy conversion. Although mechanical energy is readily generated, its utilization efficiency is generally low, and it is often regarded as an underdeveloped form of energy [85]. Beyond receiving attention in specific fields, such as mechanochemistry for organic synthesis and polymerization reactions, the potential application of mechanical energy in catalytic systems remains underexploited [86]. In this context, Tang et al. [87] developed a mechano-energy-induced CO_2_ conversion technology, utilizing a Ga-based liquid metal suspension to reduce CO_2_ to solid carbonaceous products and oxygen at near-ambient temperatures. This technology employs mechanical energy as an input to drive nanoscale Frictional electrochemistry reactions. When a Ga/silver fluoride mixture with a mass ratio of 7:1 is used as the reaction material, the CO_2_ capture and conversion process (excluding CO_2_ dissolution energy consumption) achieves 92% efficiency per ton of CO_2_, with the input energy consumption significantly reduced to 230 kilowatt-hours. It demonstrated good scalability and economic feasibility, offering a novel pathway for the green treatment of CO_2_ emissions. Mechanical energy can also be harnessed to catalyze biofuel conversion. Tang et al. [88] utilized liquid metal Ga and Ni particles as synergistic catalysts to efficiently convert renewable biofuels (such as rapeseed oil and other liquid hydrocarbons) into H_2_ and C_2_H_4_ under near-ambient temperature conditions. To gain a comprehensive understanding of the biofuel conversion process, DFT calculations were performed. Due to its smaller molecular weight facilitating computation, butyric acid was selected as a representative model for simulating the reaction of liquid hydrocarbon substances (Figure 6e). Initially, the oxygen atom of the C=O bond in the butyric acid molecule preferentially adsorbs onto the liquid Ga surface. Subsequently, the C–H bond adjacent to the C=O group undergoes spontaneous dissociation at the Ga/Ni interface. C–C bond cleavage then occurs via the *CH_2_CH_3_ intermediate, accompanied by C–H bond dissociation and C_2_H_4_ release. Notably, C–C bond cleavage possesses a relative thermodynamic advantage on the liquid Ga surface. This demonstrates that the Ga/Ni interface facilitates C–H bond activation through elemental synergy. This approach provides a sustainable method for producing H_2_ and C_2_H_4_ from renewable resources, eliminating the need for high temperatures and fossil fuels.

Furthermore, Ga-based liquid metal catalysts also exhibit outstanding catalytic performance in waste valorization. For instance, Gao et al. [89] efficiently converted polyolefin plastics (e.g., LDPE, HDPE, and PP) into narrow-distillate-range hydrocarbon oils and high-value-added olefin monomers using EGaIn and Galinstan under microwave assistance. The liquid metal was mixed with mechanically crushed polyolefin plastics, followed by reaction under microwave irradiation. The fluidity of the liquid metal and its unique chemical bond activation capabilities within the microwave field enabled the achievement of an oil yield of 85.6 wt% from polyethylene (PE) with a selectivity exceeding 50% for C2–C4 olefins; for polypropylene (PP), an oil yield of 81.0 wt% was achieved with a C2–C4 olefin selectivity of 65.3%.

## 4. Challenges and Prospects

### 4.1. Challenges

Ga-based liquid metal catalysts demonstrate significant catalytic potential due to their unique dynamic properties and play a crucial role in advancing manufacturing and technology. However, the development of this field, particularly concerning Ga-based systems, faces severe challenges. These are primarily reflected in limited alloy formulations, complex mechanistic understanding, application applicability bottlenecks, economic examination of regeneration strategies, and critical resource constraints [4]. Specifically, the following key issues require resolution:

**Barriers to Precision Control and AI-Driven Design:** Optimizing the catalytic performance of Ga-based liquid metal nanoparticles/droplets requires precise control over their size, morphology, chemical composition (particularly the distribution of dopant elements), dispersity, and wetting behavior on substrate surfaces. However, currently available regulatory approaches are limited, and their effects are difficult to accurately predict. The research and development process remains highly reliant on experience-driven and iterative trial-and-error experimental methods. This traditional strategy is inefficient and struggles to systematically explore the complex relationships between material properties and structure within high-dimensional parameter spaces. The advancement of artificial intelligence (AI) offers a potential new pathway to address this challenge. AI can learn from and integrate existing experimental and theoretical data to establish quantitative structure–activity relationships between composition/structure parameters and catalytic performance, thereby enabling high-throughput virtual design for dopant screening and ratio optimization. Furthermore, leveraging its strengths in processing complex images (e.g., microstructural features) and sequential data, AI can assist in interpreting characterization results to guide structural regulation and simulate interfacial behavior under dynamic reaction environments. Nevertheless, the practical application of AI in this field still faces critical challenges: The scarcity of high-quality datasets (due to fragmented experimental data, a lack of standardization, and the difficulty of first-principles calculations in covering actual complex interfacial environments) and the multiscale modeling gap constrain model effectiveness. Simultaneously, it is necessary to embed physical constraints (such as mass conservation and interfacial reaction kinetics equations) into the neural network architecture to overcome the limited extrapolation capability of purely data-driven models and enhance their interpretability. Significant room for development exists regarding the advancement and application of AI technology in this domain.

**Dynamic interface mechanisms and the complexity of theoretical simulations:** The surface structure (e.g., dynamically formed oxide layers), electronic state, and adsorption behavior of reaction intermediates on Ga-based liquid metal catalysts are more complex and variable than on solid catalysts. This complexity hinders the elucidation of their catalytic reaction mechanisms, impeding rational design. DFT calculations for this system face the great challenge of overcoming dynamic disorder, temperature dependence, and interface complexity. An effective research strategy requires the construction of multi-scale datasets and the development of advanced computational methods. Core datasets suitable for this system include experimental catalytic data under different reaction conditions for model validation; electronic structure data; adsorption energy data of key reactive species on the surfaces; and physicochemical data on the liquid state structure. Key computational approaches need to incorporate large-scale molecular dynamics (MD) simulations using experimentally validated machine-learning potentials or classical force fields to adequately sample the dynamic configurations; the extraction of representative snapshots from the MD trajectories for DFT structure optimization, reaction path exploration, and transition state searches and the systematic averaging of the results; and the application of enhanced sampling techniques to calculate the free energy barriers by correlating the local atomic environments to analyze the catalytic mechanism. Finally, they need to use the experimental catalytic data to close the loop to verify the reliability of the model. Specific methods include the use of AIMD or machine learning-accelerated molecular dynamics (ML-MD) for sampling and the selection of generalized functions with dispersion correction to accurately describe the interfacial adsorption.

**Application suitability bottleneck:** Although Ga-based liquid metal catalysts have demonstrated unique advantages in catalysis by virtue of their dynamic self-healing interfaces and reconfigurable active sites, their practical applications are still limited by key challenges such as long-term stability, corrosion resistance, and scalability. In terms of long-term stability, although the oxide layer on the surface of Ga can provide some protection, the oxide layer may lead to rupture under repeated catalytic cycles or extreme reaction conditions, resulting in the loss of the active components or the stripping of the interface between the liquid metal and the support material, which leads to gradual deactivation. In addition, electric field-induced ionic migration or phase separation during electrocatalysis may lead to changes in catalyst composition and performance, an effect that is particularly pronounced after prolonged operation. In terms of corrosion resistance, although Ga’s natural oxide film provides short-term protection, in electrolytes containing chloride ions or other aggressive media, the oxide layer is susceptible to localized dissolution, which can lead to pitting or homogeneous corrosion, thus destroying the integrity of the catalytic interface. Scalability is another problem that needs to be solved. The high surface tension of Ga-based liquid metals makes it difficult to be uniformly dispersed on conventional carriers, while the bulk morphology limits the exposure of active sites. Existing preparation methods are often complex and costly, and it is difficult to achieve kilogram-scale production while maintaining catalytic performance.

**Regeneration strategies and economic trade-offs:** Current regeneration strategies for Ga-based liquid metal catalysts include electrochemical methods, thermal treatments, and surface redox treatments. Although theoretically capable of restoring catalytic activity and extending material lifetime, these methods still have significant problems in practical applications. First, electrochemical regeneration requires continuous energy input, which may increase the overall process cost, especially in large-scale industrial catalytic scenarios, and it remains to be verified whether its energy efficiency provides an advantage over traditional catalyst replacement or thermal regeneration methods. Secondly, although heat treatment can remove surface oxides or reconfigure active sites, the high-temperature process may lead to the volatilization of liquid metals or structural degradation of the substrate materials, which, in turn, reduces the long-term stability of the catalytic system. In addition, the surface redox strategy often involves corrosive reagents or precise atmosphere control, which increases the process complexity and may introduce impurities and affect the catalytic selectivity. More critically, after multiple regeneration cycles, the Ga-based liquid metal may be gradually deactivated due to elemental segregation, interfacial diffusion, or irreversible phase transitions, making its regeneration effect diminish with the number of cycles. The economy, operability, and long-term reliability of the regeneration strategy are the key barriers to its practical application, which need to be optimized through systematic experiments and life cycle assessment, especially in comparison with low-cost non-regenerative catalysts, and the boundaries of their techno-economic feasibility need to be clarified.

**Resource constraints and sustainable pathways:** Ga itself has a limited natural abundance and high cost, representing two of the most fundamental constraints on the large-scale application of Ga-based liquid metal catalysts.

### 4.2. Future Research Directions

To address the above challenges, future research should focus on the following core directions to unleash its full potential:

**Material design and controllable synthesis:** Develop mild and green synthesis strategies to achieve the precise regulation of droplet size, morphology, and dispersion. Conduct an in-depth study of alloying strategies to regulate the melting point, surface tension, and electronic structure to optimize the synthesis process. Improve the carrier and interface engineering to develop carriers with special infiltration or external field responsiveness, so as to realize the stable loading of liquid metals. Construct “dynamic confinement” interfaces through strategies for confining liquid metals within or on the surface of porous materials, utilizing the physical/chemical constraints of the carrier to restrict their flow range while maximally preserving their dynamic surface characteristics, thereby achieving an optimized balance between dynamic fluidity and static stability.

**Mechanism study and performance regulation:** Conduct a systematic study of surface chemical states and reaction mechanisms, and an in-depth study of the mechanisms by which liquid metal surface oxidized layers, adsorbed species, and alloying elements affect the adsorption/activation energy barriers of specific reactants. Direct and optimize reaction paths through the precise regulation of surface chemical states. Utilizing its excellent electrical and thermal conductivity and photoresponsive properties, explore the multi-field synergistic catalytic strategies to precisely regulate the reaction process and improve the selectivity of the target products through external energy input.

**Resource recycling and sustainable development:** Deactivated Ga-based catalysts require targeted treatment based on the specific deactivation cause. Carbon deposition-induced deactivation is addressed through inert gas purging followed by temperature-programmed oxidation under controlled oxygen conditions (~450–550 °C) for coke removal, while rigorously preventing sintering. Sintering-induced deactivation is primarily managed through prevention; severely sintered catalysts necessitate replacement. Sulfur poisoning may be remediated via high-temperature hydrogen reduction, whereas poisoning by phosphorus, arsenic, or heavy metals is typically irremediable, requiring catalyst replacement and enhanced feedstock purification. Loss of active components can be partially restored by supplementing Ga precursors. Structural damage to the support renders the catalyst non-regenerable. The regeneration process must ensure safety, particularly preventing runaway temperature during coke combustion, and evaluate activity recovery, structural integrity, and economic viability. Within the recovery stage, green and efficient technologies require development: Electrochemical dissolution, magnetic separation, or density gradient separation methods should be leveraged to enhance separation efficiency, and supports designed for facile decoupling should be engineered to simplify the process. The core of high-purity Ga regeneration lies in selectively leaching Ga, followed by purification and concentration from the leachate via separation and enrichment techniques; ultimately, trace impurities are removed using ultra-high-purity metallurgical processes to achieve high-value closed-loop recycling. Residues should be prioritized for resource utilization (e.g., conversion into activated carbon or building materials); when utilization is infeasible, stabilization/solidification treatment is mandatory to ensure environmental safety.

### 4.3. Application Prospects

Having successfully addressed the aforementioned challenges and advanced research in core directions, Ga-based liquid metal catalysts, leveraging their distinct physicochemical properties including fluidity, low melting point, tunable electronic structure, and a dynamic surface, are poised to demonstrate significant application potential in the following key areas:

**Energy Field:** Ga-based liquid metal catalysts can significantly reduce the reaction temperature and energy consumption for hydrogen production and hydrogen conversion, enhancing efficiency. They provide robust catalytic support for the large-scale, economical production of green hydrogen, accelerating the development of the hydrogen industry chain. In fuel cell electrode reactions, Ga-based liquid metal catalysts demonstrate far superior activity and exceptional stability compared to traditional solid catalysts. Their liquid surface effectively prevents catalyst sintering and deactivation, significantly extending electrode lifetime and enhancing power density. This represents a critical breakthrough for advancing the commercial application of fuel cell technology and the widespread adoption of new energy sources.

**Environmental Field:** Ga-based liquid metal catalysts possess immense potential for CO_2_ activation and conversion. They can efficiently catalyze the conversion of CO_2_ into solid carbon materials or high-value-added chemicals/fuels (such as methane, methanol, and syngas derivatives). This particularly highlights their revolutionary value within the “Utilization” segment of the carbon capture, utilization, and storage (CCUS) technology chain, offering a viable technological pathway towards achieving carbon neutrality goals. Utilizing active species generated by Ga-based catalysts enables the efficient degradation of refractory organic pollutants in wastewater, providing more efficient and stable solutions for water pollution control.

**Biomedical Field:** Ga-based liquid metals serve as ideal carriers or co-catalysts. By combining with enzymes to construct enzyme–liquid metal hybrid systems, they can enhance enzyme stability (resisting denaturation), boost enzyme activity, or enable novel cascade catalytic reactions. This opens up new avenues and methods for biomanufacturing and biosensing.

## 5. Conclusions

This review summarizes the unique properties of Ga-based liquid metals and their influence on catalytic performance. It introduces the various types of Ga-based liquid metal catalysts that have emerged in the catalytic field in recent years and details their applications across diverse catalytic systems. Benefiting from exceptional thermal conductivity, fluidity (manifested as surface dynamic reconstruction), and dynamic interfacial properties, these catalysts exhibit significant advantages in both electrocatalysis and thermal catalysis. In electrocatalysis, these catalysts have been successfully applied in electrocatalytic CO_2_ reduction, electrocatalytic ammonia synthesis, electrocatalytic hydrogen production, and the electrocatalytic oxidation of alcohols, etc. In thermal catalysis, these catalysts are employed in processes such as alkane dehydrogenation, selective hydrogenation, CO_2_ reduction, and ammonia synthesis, etc. In photocatalysis, they can be used in other photocatalytic reactions such as organic matter degradation and overall water decomposition. Furthermore, Ga-based liquid metal catalysts demonstrate application potential in catalytic reactions within battery systems and reactions driven by low-grade mechanical energy. Research indicates that the dynamic surface of liquid metals can effectively prevent catalyst sintering and deactivation, significantly extending catalyst lifespan, thereby foreshadowing promising application prospects. However, critical challenges remain for this system: On one hand, the complex electronic states of liquid metals and the dynamic variations in their adsorption behavior towards reaction intermediates result in catalytic reaction mechanisms that are not yet fully elucidated. On the other hand, the low natural abundance and high cost of Ga limit the large-scale application of Ga-based liquid metal catalysts. Therefore, it is imperative to develop novel theoretical models and research methodologies to gain deeper insights into their catalytic mechanisms. Concurrently, the exploration of cost-effective liquid metal alloy systems based on abundant elements, alongside the development of efficient Ga recycling and reuse technologies, is crucial.

## Figures and Tables

**Table 1:** The melting points of common liquid metals and alloys [11,12,13,14,15].

Metal/Alloy	Element A (at%)	Element B (at%)	Element C (at%)	Melting Point [°C]
Ga	100	-	-	29.8
In	100	-	-	156.6
Sn	100	-	-	231.9
GaIn	85.8	14.2	-	15.7
GaSn	91.7	8.3	-	21.0
GaPd	4.7	95.3	-	317.0
GaInSn	67	20.5	12.5	10.8
GaInSn	75	25	10	−19.0

## Data Availability

Data are contained within the article.
